# Creating Diversified Response Profiles from a Single Quenchometric Sensor Element by Using Phase-Resolved Luminescence

**DOI:** 10.3390/s150100760

**Published:** 2015-01-05

**Authors:** Elizabeth C. Tehan, Rachel M. Bukowski, Vamsy P. Chodavarapu, Albert H. Titus, Alexander N. Cartwright, Frank V. Bright

**Affiliations:** 1 Department of Chemistry, Natural Sciences Complex, University at Buffalo, The State University of New York, Buffalo, NY 14260, USA; E-Mails: cornell.elizabeth@yahoo.com (E.C.T.); rmbukowski@gmail.com (R.M.B.); 2 Department of Electrical and Computer Engineering, McGill University, McConnell Engineering Building, 3480 University Street, Montreal, QC H3A 0E9, Canada; 3 Department of Electrical Engineering, Bonner Hall, University at Buffalo, The State University of New York, Buffalo, NY 14260, USA; E-Mails: ahtitus@buffalo.edu (A.H.T.); anc@buffalo.edu (A.N.C.)

**Keywords:** phase-resolved luminescence detection, quenchometric sensor, gaseous oxygen (O_2_) sensing

## Abstract

We report a new strategy for generating a continuum of response profiles from a single luminescence-based sensor element by using phase-resolved detection. This strategy yields reliable responses that depend in a predictable manner on changes in the luminescent reporter lifetime in the presence of the target analyte, the excitation modulation frequency, and the detector (lock-in amplifier) phase angle. In the traditional steady-state mode, the sensor that we evaluate exhibits a linear, positive going response to changes in the target analyte concentration. Under phase-resolved conditions the analyte-dependent response profiles: (i) can become highly non-linear; (ii) yield negative going responses; (iii) can be biphasic; and (iv) can exhibit super sensitivity (e.g., sensitivities up to 300 fold greater in comparison to steady-state conditions).

## 1. Introduction

**Figure 1.**
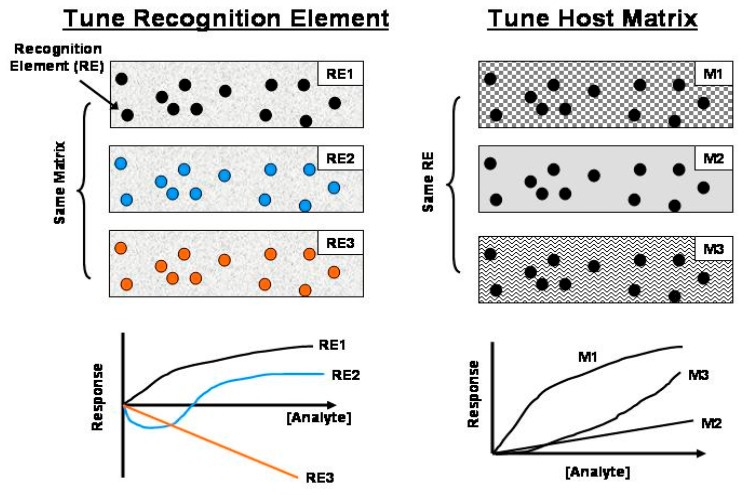
Illustration that describes traditional approaches of creating chemical sensors with a range of response profiles for the same target analyte. Three hypothetical sensor elements are shown under each approach. Each sensor yields a unique response profile for the intended target analyte that depends on the recognition element (RE*_x_*) or matrix (M*_x_*).

There has been significant effort devoted to creating optical chemical sensors for a wide variety of target analytes [1–5]. There have also been simultaneous and largely related efforts to create ensembles of sensors that act independently and provide unique responses for the same target analyte (*i.e.*, diversified responses; 1 analyte, *n* sensor elements, *n* ≫ 1)) and this concept has also been extended to simultaneous multi-analyte detection (*m* analytes, *n* sensor elements; *n* ≫ *m*) [3,6–11]. In all previous efforts, researchers have created sensor ensembles and thus diversified responses by adjusting the: (a) individual recognition elements within the analyte-permeable host matrix and/or (b) host matrix to control the partitioning of the target analyte into the host matrix and eventual interaction with the recognition element, as illustrated in Figure 1 [12–14].

In this paper, we describe a strategy for creating a continuously tunable response from a single sensor element. The approach exploits a unique feature of phase-resolved luminescence [15]. The potential of this strategy is demonstrated for a single O_2_-responsive xerogel-based sensor element as it offers a simple and well understood behavior. Consider the following scenario: (i) a single type of luminophore molecule sequestered within a quencher-permeable host matrix; (ii) these luminophore molecules emit from largely similar microenvironments; and (iii) the quencher molecules have similar accessibilities to the individual luminophore molecules. Under steady-state illumination conditions, one can write the Stern-Volmer relationship [16] given by Equation (1):

(1) $$I_{0}/I=1+K_{\text{SV}}[\text{Q}]$$

where in this expression, [Q] is the concentration of quencher, *I*_0_ and *I* represent the steady-state luminescence intensities in the absence and presence of quencher, Q, respectively, and *K*_SV_ is the Stern-Volmer quenching constant. Under dynamic quenching conditions, *K*_SV_ depends on *k*_Q_, the bimolecular quenching constant that describes the luminophore and quencher interaction and *τ*_0_, the luminophore excited-state luminescence lifetime in the absence of any quencher molecules (*i.e., K*_SV_ = *k*_Q_ τ_0_) [16].

**Figure 2.**
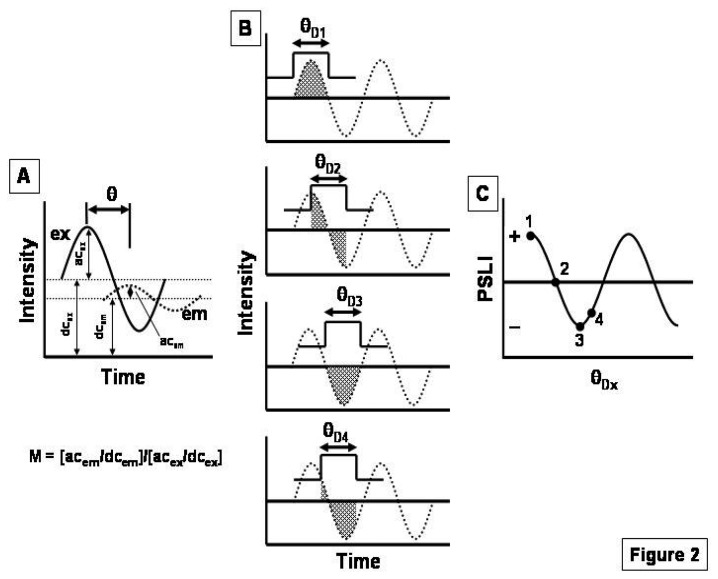
A basic phase resolved experiment. (**A**) Phase-modulation concept. Excitation (ex), emission (em), luminescence phase shift (θ), and luminescence demodulation factor (*M*) are shown; (**B**) The phase resolution experiment with the detector phase angle (θ_Dx_) set at four different values (θ_D1_ to θ_D4_). The shaded region denotes the area under the modulated emission that is integrated by the π function; (**C**) The phase-sensitive luminescence intensity (PSLI) that results from the different θ_Dx_ settings shown in (B).

If we electronically excite (ex) the luminophores within this same sensor element by using sinusoidally modulated electromagnetic radiation (Figure 2A) at frequency (*f*), the resulting emission (em) will be phase shifted (θ) and demodulated (*M*) in comparison to the excitation by an extent that depends on the luminophore excited-state lifetime (τ) [17,18]:

(2) θ=arctan(2πfτ)

(3) $$M={\left[1+{\left(2\pi f\right)}^{2}\tau^{2}\right]}^{-1/2}$$

In turn, we can write expressions for *I* and τ at any Q concentration as:

(4) $$I=I_{0}/[1+\left(k_{\text{Q}}\tau_{0}[\text{Q}]\right)]$$

(5) $$\tau =1/\left[1/\tau_{0}+k_{\text{Q}}[\text{Q}]\right]$$

If we record the luminescence from this same sensor element using a phase-sensitive detector (*i.e.*, a lock-in amplifier) (Figure 2B,C) [15], we can write an expression for the phase-sensitive luminescence intensity (PSLI) as Equation (6):

(6) $$\text{PSLI}(\theta_{\text{D}})=IM\cos(\theta_{\text{D}}-\theta )$$

where *I* denotes the steady-state luminescence intensity, *M* represents the demodulation factor, θ*_D_* is the detector (*i.e.*, lock-in amplifier) phase angle, and θ is the luminescence phase angle (all terms depend on Q). If we substitute Equations (2)–(5) into Equation (6), we can write an expression for the phase-sensitive analog of Equation (1), namely as:

(7) $${\text{PSLI}}_{0}/\text{PSLI}=\left[I_{0}M_{0}\cos(\theta_{\text{D}}-\theta_{0})\right]/\left[IM\cos(\theta_{\text{D}}-\theta )\right]$$

Equation (7) provides the key relationship between the fundamental properties of the sensor element (*i.e., k*_Q_, τ_0_, and *I*_0_) and the phase-sensitive analog of *I*_0_/*I* (*i.e.*, PSLI_0_/PSLS) as a function of [Q], θ_D_, and *f*. Inspection of Equation (7), suggests a strategy for tuning the response profiles (*i.e.*, creating diversity) from a single sensor element by adjusting θ_D_ and *f*.

## 2. Experimental Section

### 2.1. Chemical Reagents

Tris(4,7′-diphenyl-1,10′-phenanathroline) ruthenium(II) chloride pentahydrate ([Ru(dpp)_3_]^2+^) was purchased from GFS Chemicals, Inc. (Powell, OH, USA) and purified as described in the literature [19]. Tetraethyorthosilane (TEOS) and *n*-octyltriethoxysilane (C8-TEOS) were purchased from Gelest, Inc. (Morrisville, PA, USA). HCl was obtained from Fisher Scientific Co. (Pittsburgh, PA, USA). EtOH was a product of Quantum Chemical Corp. (New York, NY, USA). Deionized water was prepared to a specific resistivity of at least 18 MΩ-cm by using a Barnstead NANOpure^®^ II system.

### 2.2. Preparation of [Ru(dpp)_3_]^2+^-Doped Xerogel Sensing Films

The *sol* solutions were prepared as described elsewhere [20]. Xerogel films were formed directly onto the surface of a light emitting diode (LED, λ_max_ = 468 nm, Stanley Electric Co., Inc., (Tokyo, Japan). The LED surface was cleaned first by rinsing with 1 M NaOH then with copious amounts of deionized water and EtOH, and allowed to dry under ambient conditions. Films were formed by depositing 20 μL of sol solution on to the LED surface. Xerogel-coated LEDs were stored in the dark under ambient conditions for at least seven days before evaluation. Previous research [20] has shown that these class II xerogel-based sensor elements exhibit linear *I*_0_/*I vs.* O_2_ plots and the responses are stable for several years.

### 2.3. Instrumentation

**Figure 3.**
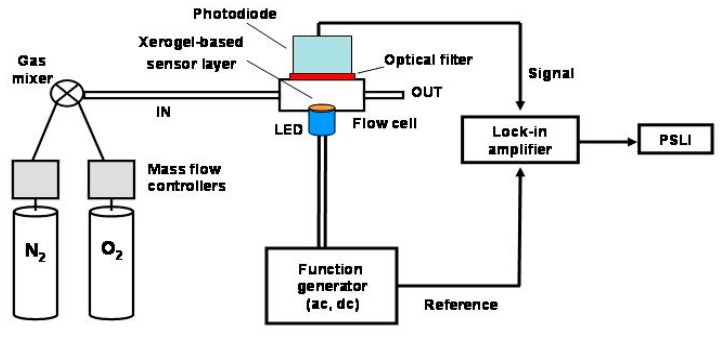
Simplified schematic of the phase-sensitive instrument used in this research. The modulation frequency (*f*) is controlled by the function generator, the detector phase angle (θ_D_) is adjusted with the lock-in amplifier, and the sample composition that reaches the sensor element is controlled by the mass flow controllers.

Figure 3 presents a simplified schematic of the phase-resolved luminescence system that was constructed for this research. A model DS345 function generator (Stanford Research Systems, Sunnyvale, CA, USA) provided the AC and DC components to drive the LED. The function generator output also served as the reference input to a Stanford Research Systems (model SR830) lock-in amplifier. The xerogel-coated LED was enclosed within an optically transparent housing with an inlet/outlet for gas flow. In the current experiments, the quencher was gaseous O_2_. The emission was monitored through a 570 nm long pass optical filter (Oriel, Irvine, CA, USA) and detected by a 1 MHz bandwidth silicon photodiode (Thorlabs, Inc., Newton, NJ, USA). The photodiode output signal was directed to the lock-in amplifier input. The lock-in amplifier served to record the phase sensitive luminescence intensity (PSLI) at a given detector phase angle setting (θ_D_).

In a typical experiment, we adjust the excitation modulation frequency (*f*) to a given value (20 or 50 kHz) and increment θ_D_ in 22.5° steps from 0° to 180°. We then systematically adjust the environment surrounding the sensor from pure N_2_ to pure O_2_ by using a pair of precision mass flow controllers (model PC1NP1U1V_1A, Sensirion Inc. (Westlake Village, CA, USA). Equilibrium is evident when the luminescence intensity remains constant to within ±2%. There was no detectable sensor response hysteresis or photo-bleaching. The O_2_ concentrations were accurate to ±0.8% between 10% and 100% full scale and to ±0.08% below 10% full scale.

The θ_D_-, *f*-, and O_2_-dependent phase-resolved Stern-Volmer plots were then constructed. Sensors were prepared in triplicate and replicates were prepared using at least three reagent batches on different days. All experiments were performed in at least triplicate over the course of several months. Results are reported as the mean for all measurements under a given set of conditions ± one standard deviation.

## 3. Results and Discussion

**Figure 4.**
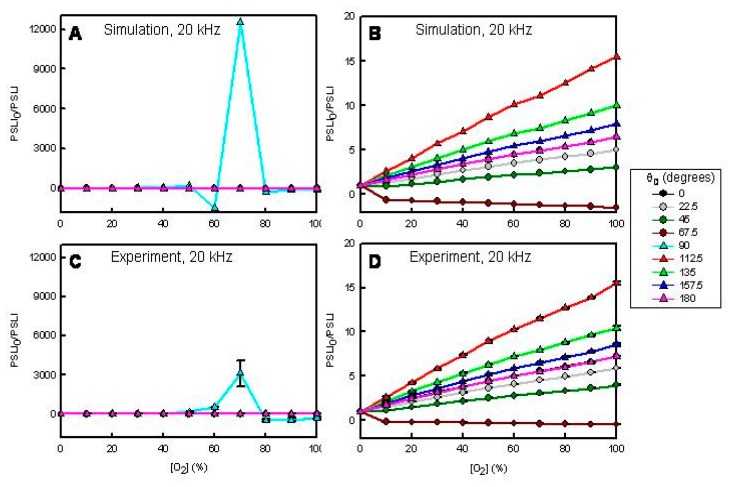
Simulated (**A**,**B**) and experimental (**C**,**D**) O_2_-dependent, phase sensitive Stern-Volmer plots for a [Ru(dpp)_3_]^2+^-doped C8-TEOS/TEOS-based xerogel film at *f* = 20 kHz. In the simulations τ*_0_* = 5.0 μs and *K*_SV_ = 0.1 O_2_%^−1^. (**B**,**D**) θ*_D_* = 90° data omitted.

**Figure 5.**
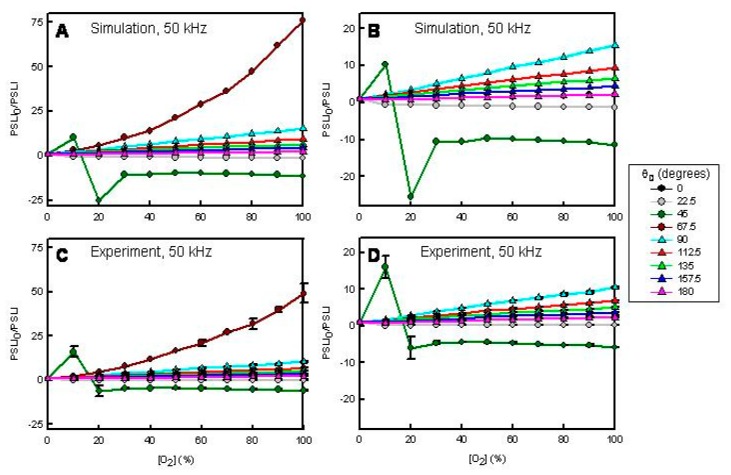
Simulated (**A**,**B**) and experimental (**C**,**D**) O_2_-dependent, phase sensitive Stern-Volmer plots for a [Ru(dpp)_3_]^2+^-doped C8-TEOS/TEOS-based xerogel film at *f* = 50 kHz. In the simulations τ*_0_* = 5.0 μs and *K*_SV_ = 0.1 O_2_%^−1^. (**B**,**D**) θ*_D_* = 67.5° data omitted.

Figures 4 and 5 present simulated (A and B) and experimental (C and D) results for the sensor film when modulated at 20 and 50 kHz, respectively, as a function of O_2_ (quencher) concentration and θ_D_. In the simulations we assumed: τ_0_ = 5.0 μs and *K*_SV_ = 0.1 O_2_%^−1^. Figures 4A and 5A show the simulated responses at each θ_D_. Figures 4B and 5B show the simulated response with the highest responses (θ_D_ = 90° in Figure 4A, θ_D_ = 67.5° in Figure 5A) removed. Figures 4C and 5C show the experimental responses at each θ_D_. Figures 4D and 5D show the experimental responses with the highest responses (θ_D_ = 90° in Figure 4C, θ_D_ = 67.5° in Figure 5C) removed.

Inspection of these data sets shows several interesting results. First, the simulations and experiments agree very well. Second, the response profiles depend on *f* and θ*_D_*. Thus, one can tune the sensor response and sensitivity simply by adjusting *f* or θ*_D_*. In all previous sensor research, there was no provision for achieving a diverse response from a single sensor element (*cf.*, Figure 1). Third, the response profiles are sometimes non-linear. In a steady-state experiment this sensor element exhibits a linear response with a *K*_SV_ of ∼0.1 O_2_%^−1^ [20]. Fourth, there are conditions when the phase-resolved Stern-Volmer plots exhibit negative going responses due to negative phase shift change. Fifth, there are conditions when the phase-resolved Stern-Volmer plots exhibit biphasic response profiles. Finally, there are conditions when the response (PSLI_0_/PSLI) is ultra-sensitive. For example, in our previous work, the *I*_0_/*I* for 70% O_2_ is ∼8 for this sensor element [20]. In Figure 4C we show a reproducible response in excess of 3000 at 20 kHz with a θ_D_ of 90°. Similarly, at 50 kHz with a θ*_D_* of 67.5° we see a PSLI_0_/PSLI at 100% O_2_ of ∼50 whereas the *I*_0_/*I* at 100% O_2_ is ∼10 for this same sensor [20].

## 4. Conclusions

We report on a simple and new approach for creating diversified sensor responses from a single sensor element. The approach is not only limited to quenchometric-based sensing, but also will work under any situation where there is a change in a luminophore excited state lifetime caused by the target analyte [21–25]. Phase-sensitive detection also leads to unique analyte-dependent response profiles that are not realized under previously used detection formats. These diversified responses are ideal for training artificial neural networks (ANNs) to identify patterns and features for improving the accuracy and precision of analyte concentration determinations [26]. This strategy of creating a diversified response from a single sensor element could also enable novel miniaturized sensor systems with built-in self-testing when integrated with a tunable multi-frequency phase fluorometric spectroscopic systems [27].
